# Worldwide long-term trends in the incidence of nonalcoholic fatty liver disease during 1990–2019: A joinpoint and age-period-cohort analysis

**DOI:** 10.3389/fcvm.2022.891963

**Published:** 2022-09-12

**Authors:** Wentao Wu, Aozi Feng, Wen Ma, Daning Li, Shuai Zheng, Fengshuo Xu, Didi Han, Jun Lyu

**Affiliations:** ^1^Department of Clinical Research, The First Affiliated Hospital of Jinan University, Guangzhou, China; ^2^School of Public Health, Xi'an Jiaotong University Health Science Center, Xi'an, China

**Keywords:** nonalcoholic fatty liver disease, epidemic trend, incidence, age-period cohort model, prevention strategy

## Abstract

**Background:**

Non-alcoholic fatty liver disease (NAFLD) was previously a neglected disease that is now becoming a worldwide pandemic. A better understanding of its incidence and long-term trends will help to increase public awareness of the disease and the development of future prevention strategies.

**Methods:**

The incidence rates of NAFLD during 1990–2019 were collected from the Global Burden of Disease Study 2019 database according to the following parameters: sex, age, socio-demographic index, and geographical region. Estimated annual percentage changes and joinpoint models were used to assess the long-term trend of NAFLD, and an age-period-cohort model was used to assess the extents of the age, period, and cohort effects.

**Results:**

Adult males, postmenopausal females, Latin American populations, and people in developing countries had a high risk of developing NAFLD. The joinpoint model indicated a new trend of increasing NAFLD incidence in 2005. Age was a risk factor affecting NAFLD incidence, with this effect increasing in more-recent periods. Younger birth cohorts had lower risks of NAFLD.

**Conclusions:**

Recent prevention measures for NAFLD have achieved good initial results. However, it remains a high priority to increase the public awareness of this condition, develop its diagnostic criteria, identify cost-effective screening methods, and seek policy support to act against NAFLD, which will be a major public health problem in the future.

## Introduction

Chronic liver disease (CLD) is a major cause of mortality, morbidity, and health care resource utilization worldwide (1). An important contributor to its increased disease burden is the worldwide pandemic of nonalcoholic fatty liver disease (NAFLD), which is the most common form of CLD and the cause of end-stage liver disease in many regions (2–4).

NAFLD refers to a clinicopathological syndrome caused by excessive fat deposition in liver cells after excluding alcohol and other clear liver damage factors [fat accumulation, drugs, genetic diseases, and viral infections e.g. (5)]. Early NAFLD cases are rare and have not caused widespread concern. Since 1990, with the worldwide pandemic of obesity and type 2 diabetes, NAFLD has evolved from a previously largely unknown liver disease to the most prominent CLD worldwide (6). At the same time, overnutrition, and sedentary lifestyles induced by rapid worldwide urbanization may further exacerbate the increasing NAFLD incidence (7, 8). In addition to the disease burden associated with liver disease, NAFLD shares metabolic risk factors with multiple cardiovascular diseases (CVD), and there is evidence that NAFLD is strongly associated with increased risk of major CVD events such as cardiomyopathy, heart valve disease, and primary arrhythmias (9). Unlike other highly prevalent diseases, NAFLD is ignored by most national strategies and policies for chronic non-communicable diseases (10, 11). In fact, there is a huge gap between the enormous burden of NAFLD and the current understanding of the disease.

In the face of this emerging pandemic, understanding, and predicting long-term NAFLD trends can provide clues to the role of risk factors in its etiology. The Global Burden of Disease (GBD) study provides incidence data on NAFLD from 204 countries and regions during 1990–2019; through these indicators, it can further evaluate long-term NAFLD incidence trends and make predictions about future trends. In the present study, we conducted various subgroup analyses to better understand the NAFLD incidence according to age group, sex, geographical regions, and socio-demographic index (SDI). We also used joinpoint regression and age-period-cohort (APC) models to evaluate the NAFLD incidence trend over time and the impacts of age, period, and cohort effects on the disease burden during 1990–2019. These results will help the rational allocation of limited medical resources, and it will also help decision-makers to specify policies according to local conditions.

## Methods

The data source of this study was the Global Health Data Exchange (GHDx) query tool (http://ghdx.healthdata.org/gbd-results-tool) that was established by and improved on in the GBD study. The GBD study has continuously produced and updated worldwide disease burden data since 1990, which are freely available to the public. Up-to-date data on the burden of human disease in the 29 years of 1990–2019 can be obtained for free using the GHDx query tool (12). The GHDx data were derived from various sources, and includes relevant population registration documents, verbal autopsy data, death health test data, demographic survey data, and data provided by medical institutions in various regions of the world (13). General estimation methods for GBD data have been widely reported previously (13, 14). Data on the NAFLD burden were reasonably estimated using the DisMod-MR 2.1 model after a PubMed search and a systematic literature review (15, 16). We customize and explore disease burden data through GHDx. For example, in order to obtain the age-standardized incidence rate of NAFLD worldwide in 2019, we selected “Cause of death or injury” in the drop-down key of “GBD Estimate,” “Incidence” in the drop-down key of “Measure,” “Rate” in the drop-down key of “Metric,” “Total Burden related to NAFLD” in the drop-down key of “Cause,” “Location” in the drop-down key of “Global,” “Age” in the drop-down key of “Age-standardized,” and finally click “Year” in the drop-down key of “2019.” According to the above process of NAFLD burden data extraction, we can obtain the incidence data of different genders, ages and years in 5 SDI regions, 21 geographical regions and 204 countries and territories.

### Statistical analysis

Age-standardized rates (ASRs) and estimated annual percentage changes (EAPCs) were used to evaluate the NAFLD incidence trends over the 29-year period of 1990–2019. EAPC is an indicator used to measure the ASR change trend within a specified time interval in time series data, and is calculated as EAPC=100×(Exp(β) −1), where β refers to the ASR trend (17). To evaluate the ASR change trend, it is necessary to calculate the EAPC value and its 95% CI. Specifically, when the EAPC value and the lower 95% CI limit are both positive, ASR is considered to be increasing, whereas when the EAPC value and upper 95% CI limit are both negative, ASR is considered to be decreasing. Gaussian regression and Pearson's correlation coefficients were used to evaluate the association between ASR and SDI in 2019. Joinpoint regression models were used to assess long-term trends and critical time points for NAFLD (18). The joinpoint regression model is often used to fit the trend of the incidence of the target disease in the observed period. The principle is to calculate the residual square error between the estimated value and the true value by the least square method, so as to judge the turning point of the trend of the rate change. When the joinpoint is 0, the image of the regression equation is a straight line, and the joinpoint increases with the change of the slope (19). When the EAPC calculated by the model is higher than 0, the incidence rate can be considered to have increased each year during an observation period, whereas the incidence rate is considered to have decreased each year during an observation period if the EAPC is lower than 0. This kind of quantitative trend analysis can avoid the bias from only obtaining conclusions through observation. The APC model, which was based on Poisson distribution, can be used to analyze the relationships between disease occurrence and ages, periods, and birth cohorts. The general formula of the model is as follows:


$$\begin{array}{l} M_{\text{ij}}=D_{\text{ij}}/P_{\text{ij}}=\mu +\alpha_{\text{i}}+\beta_{\text{j}}+\gamma_{\text{k}+}\;\varepsilon_{\text{ijκ}} \end{array}$$

where i represents different age groups, j represents different periods, M, D, and P are the incidence rate, number of new cases, and total number of exposed persons, respectively, α and β are coefficients of the age and period groups, respectively, γ_k_ is the coefficient of the κth cohort group, μ is the intercept, and ε_ijκ_ is the random sampling error (20). It is clear that since there is a linear correlation among age, period, and queue (queue = age-period), the solution of the model is not unique, which is an unsolvable problem of APC models (21, 22). The endogenous factor method is a way to generate model parameter estimates using vector space projection (23, 24). This method was used in the present study to address that unsolvable problem.

When assessing incidence trends in ages, periods, and cohorts, age and period must be segregated, and models require age and period intervals to be consistent. The commonly used classification standard was adopted in the present study, with 5-year intervals for both age groups and observation periods (25). In order to include as many observation periods as possible to determine long-term trends in incidence, six time points were selected during 1990–2019: 1994, 1999, 2004, 2009, 2014, and 2019. Since people younger than 15 years and people older than 90 years were classified as separate populations in the GBD study, people aged 15–89 years were selected as the research subjects for the age-cohort model in this study. We can therefore assess the age, period, and cohort effects.

All statistical tests were two-sided, and *P* < 0.05 was considered statistically significant. R software (version 3.5.1) and Stata software (version 15.0) were used to calculate EAPC, the map of the disease burden, Gaussian regression, and Pearson's coefficient to establish the joinpoint and APC models.

## Results

In 2019, the worldwide incidence of NAFLD was 172,329.57 millions, corresponding to twice that in 1990. During those 29 years, the age-standardized incidence rate (ASIR; per 100,000 persons) of NAFLD had a stable trend.

### Differences in ASIR between sexes

**Figure 1:**
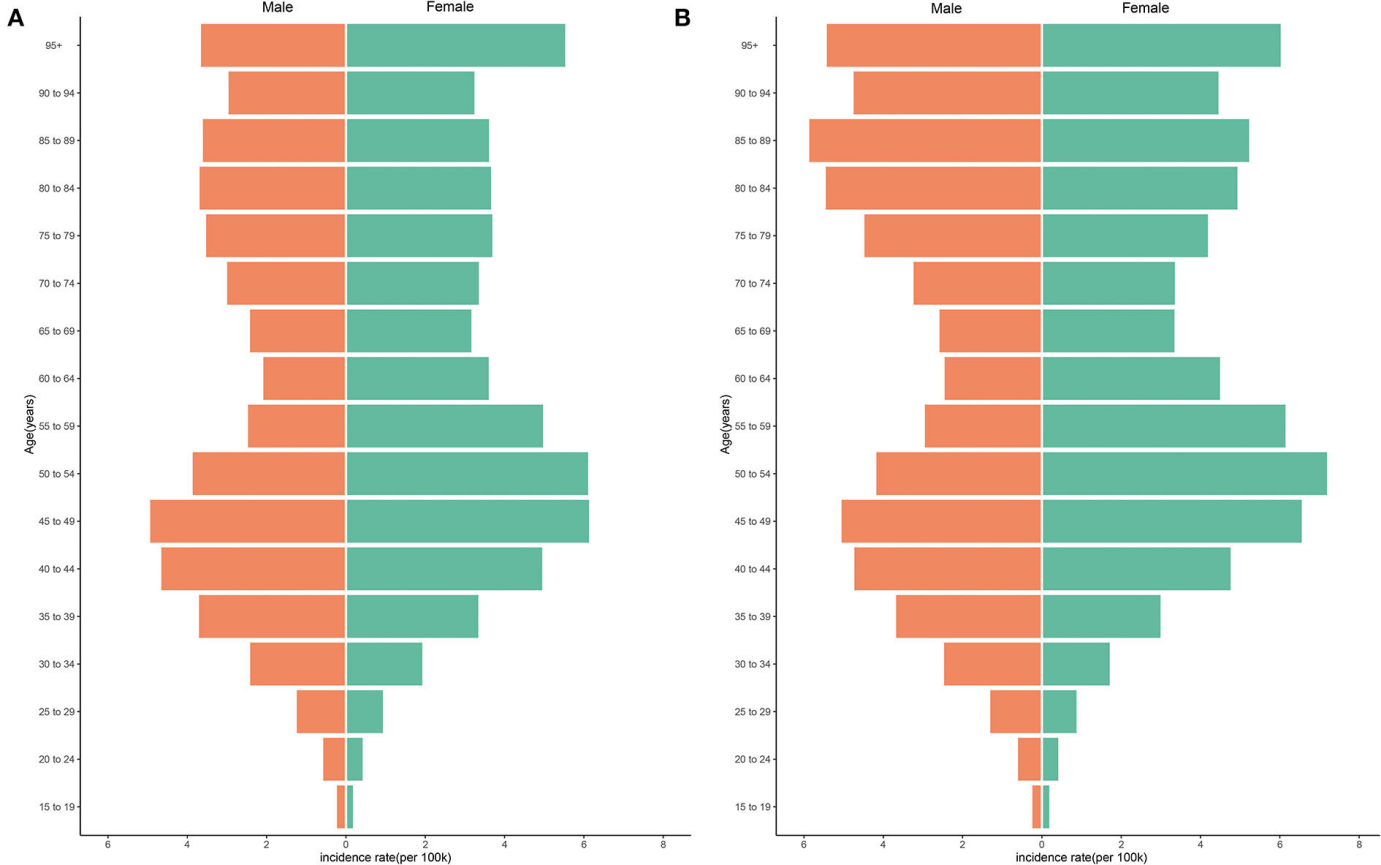
Difference in ASIR of NAFLD between genders in 1990 and 2019. **(A)** ASIR in 1990. **(B)** ASIR in 2019.

The ASIRs of the two sex groups have remained stable over the 29-year study period (Table 1). It was especially interesting that for the data collected during 1990–2019, the ASIR of males younger than 45 years was generally higher than that of the respective females, while the ASIR of females older than 45 years was often higher than that of the respective males. In 1990, the highest ASIR for females was among those aged 45–49 years and lowest among those aged 15–19 years, and the highest for males was among those aged 85–89 years and lowest among those aged 15–19 years. In 2019, the highest ASIR for females was among those aged 50–54 years and the lowest was among those aged 15–19 years, and the highest for males was among those aged 85–89 years and lowest among those aged 15–19 years (Figure 1A). It is worth noting that in 1990, the ASIRs of different age groups had a unimodal distribution trend, and the highest ASIRs for both males and females were all distributed in the three groups of those aged 40–44, 45–49, and 50–54 years. In 2019, ASIR had a bimodal distribution trend. Except for those aged 40–54 years, the ASIRs of males and females all had increasing trends for those aged 80–84, 85–89, 90–94 years, and ≧95 years (Figure 1B).

**Table 1:** The incident cases and ASIR in 1990 and 2019 and its temporal trends.

**Characteristics**	**1990**		**2019**		**1990–2019**
	**Count**	**Age-standardized rate(per 100 000)**	**Count**	**Age-standardized rate(per 100 000)**	**EAPC**
	**Both (95%CI)**	**Both (95%CI)**	**Both (95%CI)**	**Both (95%CI)**	**Both (95%CI)**
Global	88176.63 (62303.78 to 128318.53)	1.94 (1.38 to 2.77)	172329.57 (125775.23 to 243639.95)	2.08 (1.52 to 2.93)	0.1 (−0.04 to 0.23)
**Sex**
Female	47192.25 (33497.32 to 66220.97)	2.08 (1.48 to 2.93)	94459.58 (67936.07 to 132204.84)	2.23 (1.6 to 3.12)	0.12 (−0.02 to 0.26)
Male	40984.38 (28478.92 to 61166.87)	1.79 (1.27 to 2.56)	77869.99 (55777.26 to 111242.86)	1.93 (1.4 to 2.74)	0.06 (−0.07 to 0.2)
**Socio-demographic index**
High SDI	17351.5 (12126.28 to 25237.81)	1.87 (1.3 to 2.75)	28915.26 (21907.95 to 39199.27)	2.16 (1.58 to 3.06)	0.31 (0.22 to 0.41)
High-middle SDI	23487.76 (16685.69 to 34068.48)	2.08 (1.48 to 2.94)	36644.62 (26064.47 to 53096.92)	1.99 (1.41 to 2.92)	−0.28 (−0.44 to −0.12)
Low SDI	3867.22 (2533.51 to 5741.97)	1.28 (0.85 to 1.89)	10509.68 (7246.8 to 15577.89)	1.5 (1.04 to 2.19)	0.41 (0.28 to 0.54)
Low-middle SDI	10954.87 (7507.1 to 15988.12)	1.4 (0.99 to 2.00)	26644.87 (18850.8 to 38650.77)	1.66 (1.19 to 2.39)	0.4 (0.15 to 0.64)
Middle SDI	32463.2 (22799.18 to 46322.37)	2.49 (1.8 to 3.44)	69672.19 (50155.81 to 98086.14)	2.58 (1.9 to 3.57)	0.04 (−0.14 to 0.21)
**Region**
Andean Latin America	894.85 (615.13 to 1311.75)	3.44 (2.39 to 4.97)	3418.15 (2344.77 to 4867.31)	5.62 (3.86 to 7.97)	1.7 (1.56 to 1.83)
Australasia	305.89 (208.08 to 436.32)	1.4 (0.95 to 2.00)	650.98 (484.77 to 867)	1.74 (1.27 to 2.4)	1.02 (0.86 to 1.18)
Caribbean	793.56 (557.02 to 1125.29)	2.74 (1.95 to 3.87)	1472.5 (1007.5 to 2130.5)	2.9 (2.00 to 4.22)	0.08 (−0.06 to 0.22)
Central Asia	986.92 (646.18 to 1486.27)	1.81 (1.18 to 2.74)	3963.11 (2663.11 to 5829.81)	4.19 (2.87 to 6.08)	3.17 (3.06 to 3.28)
Central Europe	1972.4 (1429.31 to 2785.77)	1.45 (1.04 to 2.06)	1970.46 (1388.25 to 2823.72)	1.30 (0.89 to 1.93)	−0.2 (−0.31 to −0.1)
Central Latin America	7341.95 (4791.22 to 11105.8)	6.11 (4.03 to 9.06)	17862.62 (12133.52 to 25983.57)	6.88 (4.7 to 9.98)	0.64 (0.53 to 0.76)
Central Sub-Saharan Africa	361.86 (233.71 to 557.46)	1.09 (0.70 to 1.69)	1201.81 (770.87 to 1821.47)	1.37 (0.89 to 2.1)	0.56 (0.37 to 0.75)
East Asia	28171.11 (20355.04 to 39052.1)	2.67 (1.96 to 3.65)	42266.81 (29933.07 to 59576.4)	2.10 (1.51 to 2.93)	−1.26 (−1.64 to −0.88)
Eastern Europe	2855.48 (1806.02 to 4578.11)	1.18 (0.74 to 1.88)	4929.18 (3042.07 to 7942.38)	2.04 (1.22 to 3.32)	2.34 (2.19 to 2.49)
Eastern Sub-Saharan Africa	1895.61 (1184.04 to 2889.93)	1.91 (1.18 to 2.93)	5350.42 (3490.45 to 8069.7)	2.31 (1.48 to 3.5)	0.56 (0.47 to 0.66)
High-income Asia Pacific	2837.51 (2177.33 to 3778.84)	1.39 (1.07 to 1.84)	4589.44 (3635.91 to 5805)	1.27 (0.99 to 1.65)	−0.59 (−0.88 to −0.29)
High-income North America	5970.96 (3937.27 to 9336.86)	1.9 (1.24 to 2.95)	11217.6 (8097.54 to 15711.05)	2.58 (1.81 to 3.79)	0.81 (0.61 to 1.02)
North Africa and Middle East	3861.58 (2738.91 to 5469.58)	1.95 (1.37 to 2.76)	15287.61 (10768.37 to 21692.29)	3.01 (2.14 to 4.22)	1.65 (1.54 to 1.76)
Oceania	36.43 (24.55 to 52.97)	0.81 (0.58 to 1.13)	84.18 (57.6 to 120.77)	0.82 (0.6 to 1.13)	−0.01 (−0.09 to 0.06)
South Asia	5627.65 (3504.46 to 8734.56)	0.76 (0.5 to 1.12)	14855.43 (9902.06 to 22530.13)	0.91 (0.62 to 1.33)	0.34 (0.02 to 0.67)
Southeast Asia	7319.1 (4892.84 to 10800.93)	2.15(1.44 to 3.14)	17475.62 (12608.07 to 24540.15)	2.54 (1.86 to 3.52)	0.55 (0.49 to 0.62)
Southern Latin America	912.95 (580.32 to 1391.53)	1.96 (1.24 to 2.99)	1982.35 (1293.06 to 3012.53)	2.65 (1.72 to 4.06)	0.98 (0.91 to 1.05)
Southern Sub-Saharan Africa	510.68 (345.73 to 741.25)	1.48 (1.00 to 2.16)	1063.34 (775.73 to 1465.98)	1.61 (1.20 to 2.17)	0.01 (−0.2 to 0.22)
Tropical Latin America	2243.24 (1377.64 to 3495.67)	1.77 (1.08 to 2.72)	4404.23 (2791.59 to 6575.8)	1.72 (1.10 to 2.56)	−0.24 (−0.41 to −0.07)
Western Europe	11664.15 (7929.39 to 17367.33)	2.71 (1.82 to 4.05)	13552.43 (9717.97 to 19415.52)	2.46 (1.70 to 3.62)	−0.43 (−0.56 to −0.3)
Western Sub-Saharan Africa	1612.75 (1044.97 to 2415.58)	1.52 (1.00 to 2.24)	4731.29 (3131.06 to 6970.26)	1.83 (1.22 to 2.66)	0.53 (0.43 to 0.62)

### Differences in ASIR among different SDI quintiles

**Figure 2:**
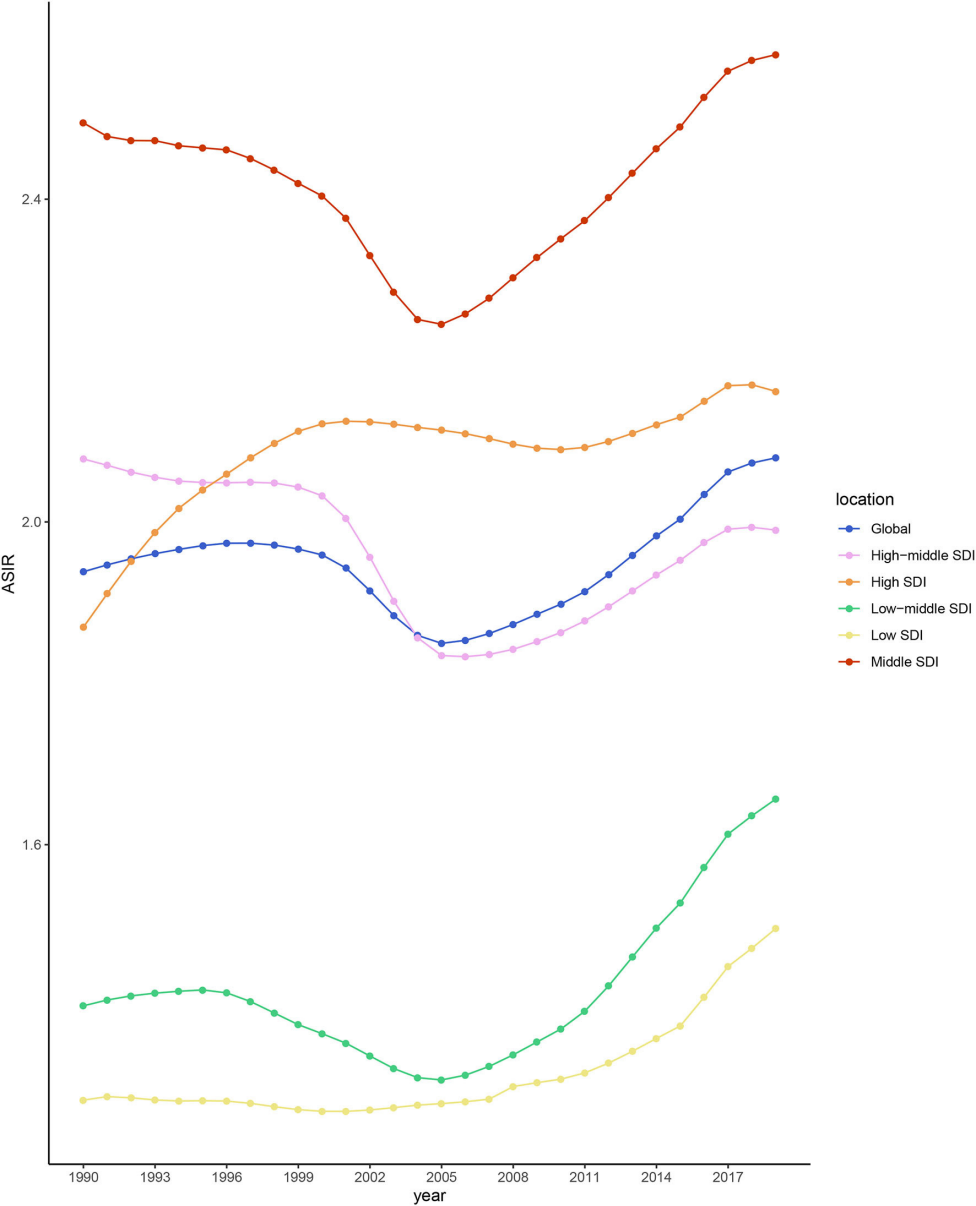
ASIR differences between different SDI subgroups from 1990 to 2019.

**Figure 3:**
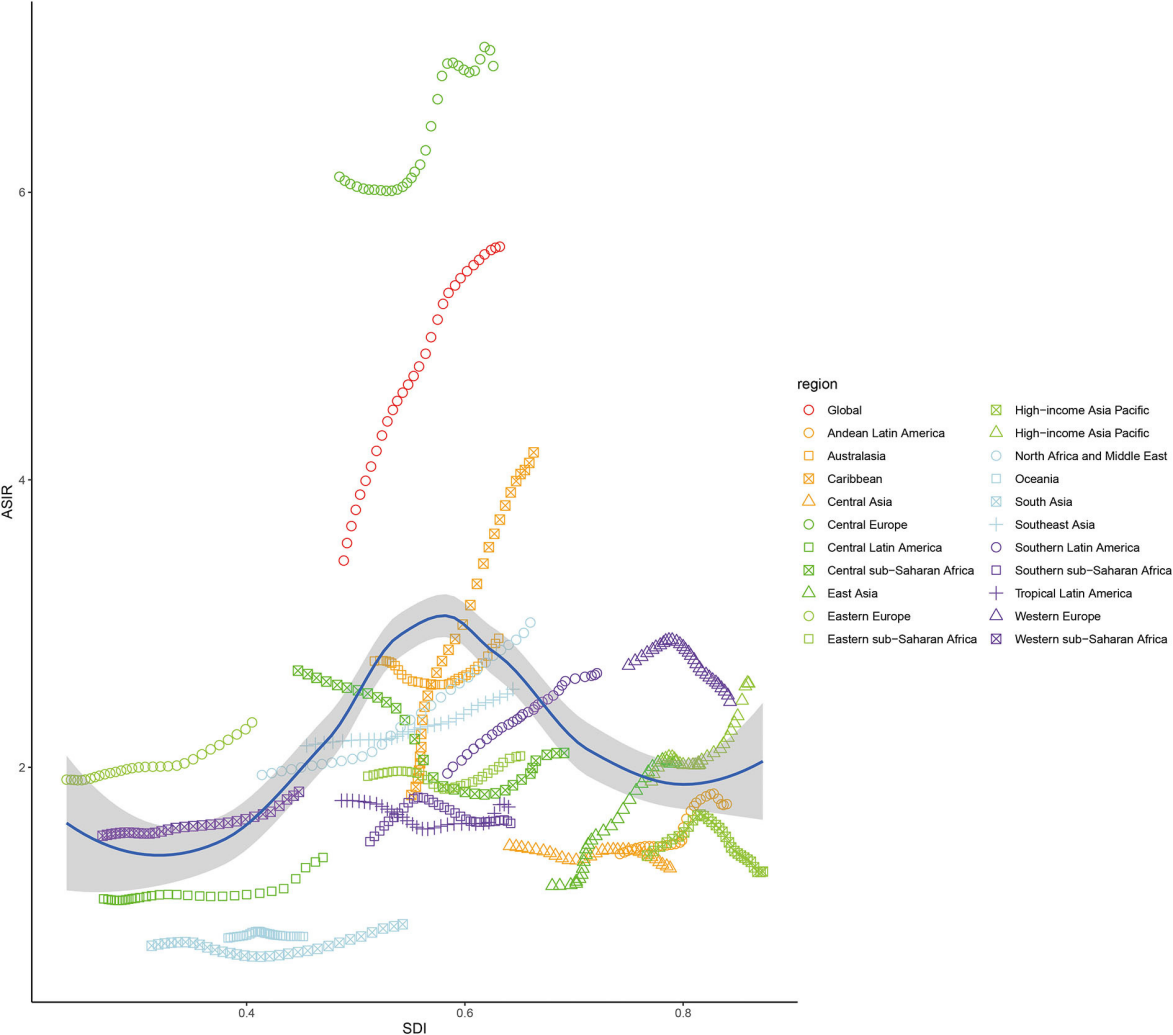
Correlation between SDI index and ASIR.

During 1990–2019, the ASIR of NAFLD was consistently the highest in the middle-SDI group and lowest in the low-SDI group (Table 1 and Figure 2). The ASIRs of the high-, low-, and low-middle-SDI groups had increasing trends, the high-middle-SDI group had a decreasing trend, and the middle-SDI group had a stable trend. In terms of the long-term trend, the ASIRs of all groups except the high-middle-SDI group exhibited a trend that initially decreased then increased, with the turning point being around 2005. In contrast, the trend of the high-middle-SDI group was slowly increasing (Figure 2). As of 2019, the fastest-increasing group was the middle-SDI group and the slowest was the high-SDI group. Figure 3 illustrates the association between ASIR and SDI in 2019. ASIR and SDI had an inverted U-shaped association. When SDI <0.6 there was a positive correlation, and when SDI >0.6 there was a negative correlation.

### Differences in ASIR among different regions

**Figure 4:**
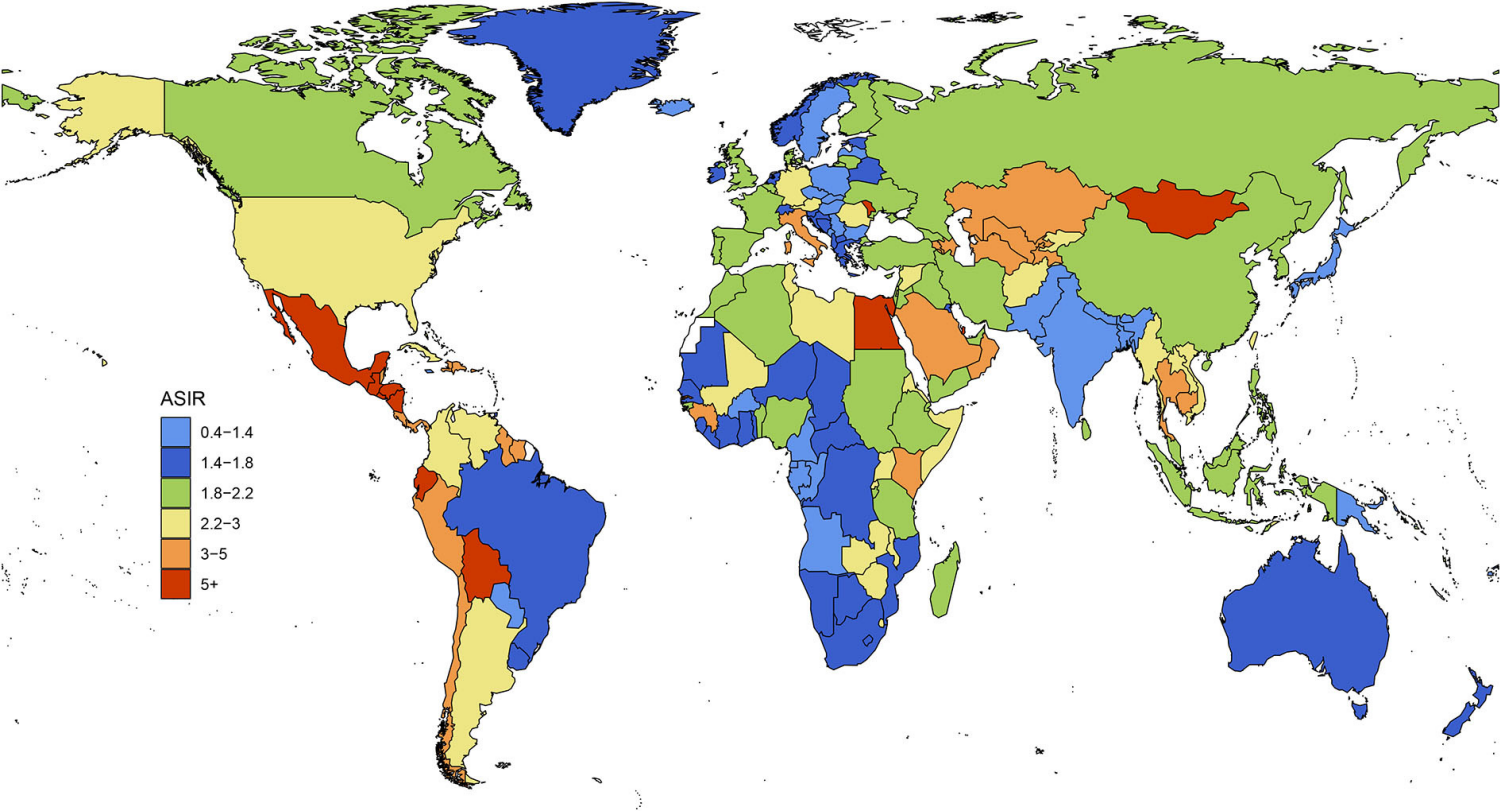
Differences in ASIR between different geographical regions.

In 1990, the three regions with the highest ASIRs were Central Latin America, Andean Latin America, and the Caribbean, among which it was clearly highest in Central Latin America. The three regions with the lowest ASIRs were South Asia, Oceania, and Central sub-Saharan Africa. In 2019, the three regions with the highest ASIRs were Central Latin America, Andean Latin America, and Central Asia, among which it was clearly highest in Central Latin America. The three regions with the lowest ASIRs were Oceania, South Asia, and high-income Asia Pacific. During those 29 years, the ASIRs of 13 regions increased, with Eastern Europe having the fastest increase; ASIRs decreased in 5 regions, with East Asia having the fastest decrease; while the trend remained stable in 3 regions: the Caribbean, Oceania, and Southern sub-Saharan Africa (Figure 4).

### Joinpoint regression model results

**Figure 5:**
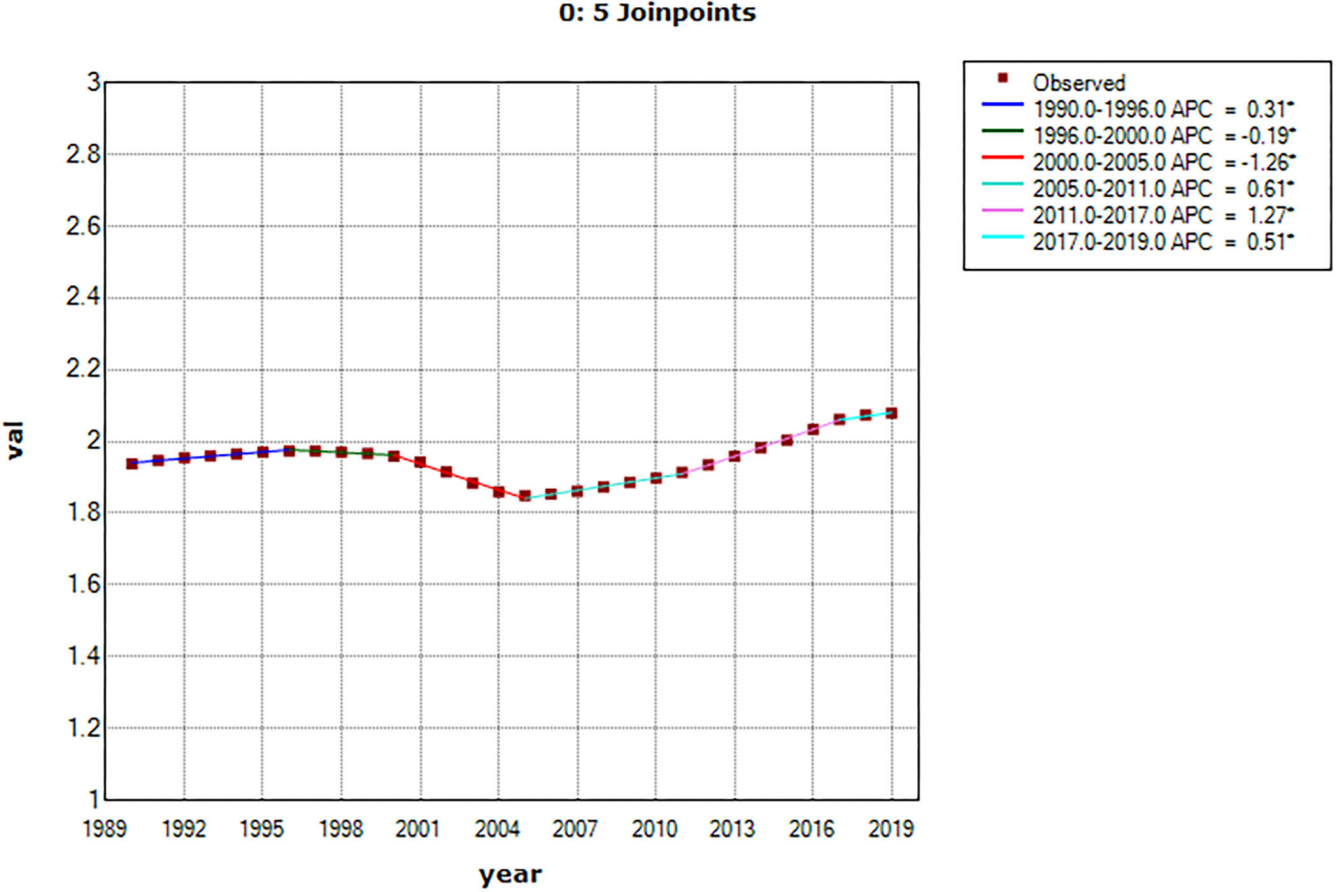
The results of joinpoint model and the long-term trend of ASIR.

During the 29-year observation period, the worldwide NAFLD incidence trend underwent five transitions, and the results of joinpoint regression model indicated five joinpoints. Based on the dates of these joinpoints, the 29 years can be divided into 6 different periods: 1990–1996, 1996–2000, 2000–2005, 2005–2011, 2011–2017, and 2017–2019 (Figure 5). Comparing the value of 0 and annual percentage changes indicates that the worldwide ASIR of NAFLD had a trend that initially decreased and then increased during the observation period.

### Age, period, and cohort trends, and the APC model results for the worldwide NAFLD incidence

**Figure 6:**
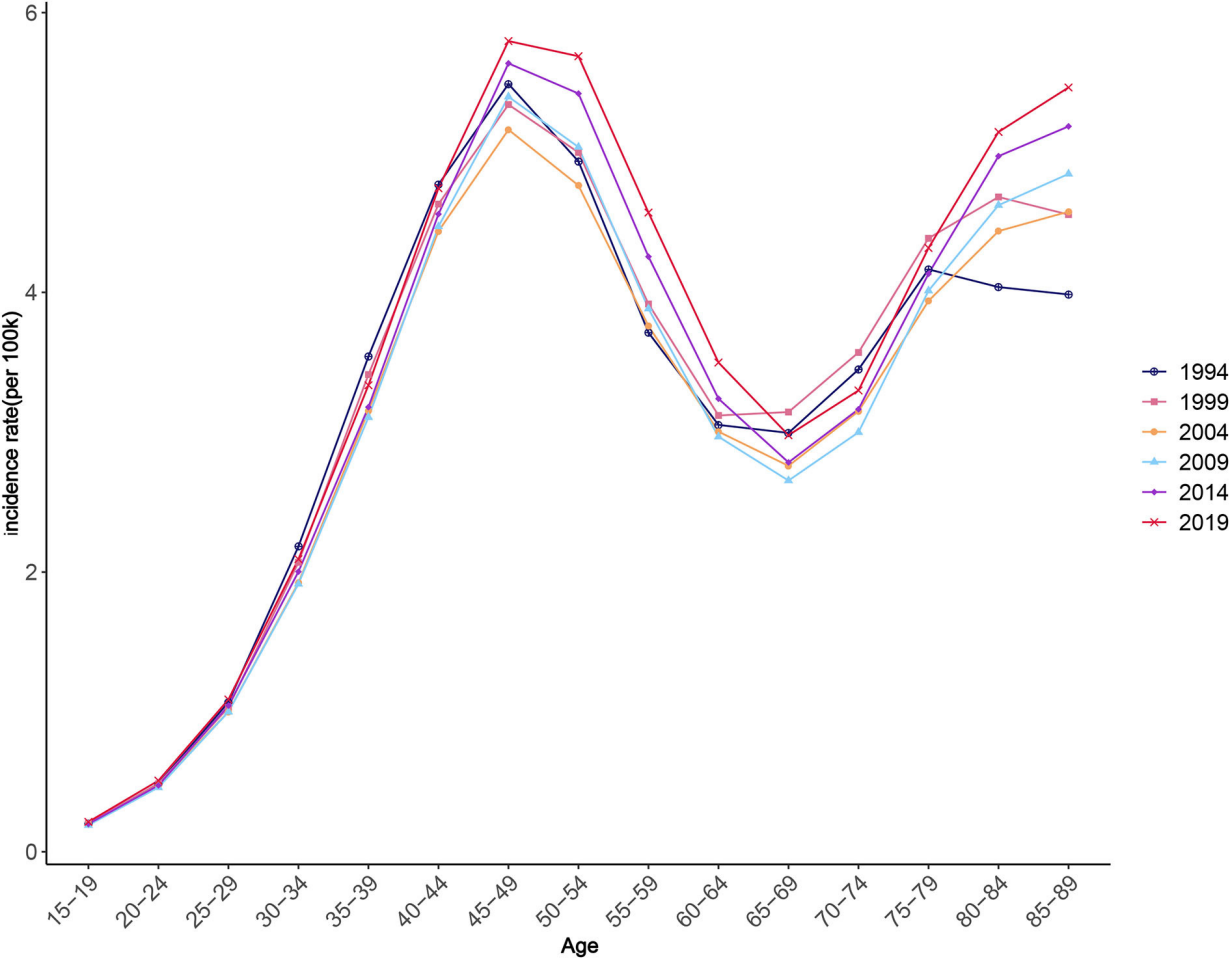
Age trends in NAFLD incidence.

Figure 6 illustrates the change trend of ASIR for worldwide NAFLD during the observation period. As can be seen from the changing trend of the graph, NAFLD incidence significantly increased then decreased with age during 1990–2019, and then increased again. ASIR exhibited a rapidly increasing trend between each observation point as age increased for those younger than 45–49 years, a decreasing trend for those aged 45–49 years to 65–69 years, and an increasing trend for those aged over 65–69 years. The APC model results further strengthened the evidence of this trend. From the quantitative results of the effect coefficients of different age groups, the age effect was largest in those aged 45–49 years, with a coefficient of 0.811, and smallest in those aged 15–19 years, with a coefficient of −2.15, both with statistical significance. These results suggest that the age group with the highest risk was 45–49 years, and that with the lowest risk was 15–19 years. The increased NAFLD risk due to the age effect was mostly concentrated among people older than 30 years (Table 2).

**Table 2:** The results of age-period-cohort model.

**Effect**	**Effect coefficient**	***P***
**Age**
15–19	−2.146 (−3.91 to −0.382)	0.017
20–24	−1.325 (−2.42 to −0.23)	0.018
25–29	−0.62 (−1.431 to 0.191)	0.134
30–34	−0.016 (−0.199 to −0.113)	0.047
35–39	0.406 (−0.144 to 0.955)	0.148
40–44	0.688 (0.198 to 1.178)	0.006
45–49	0.811 (0.358 to 1.264)	0
50–54	0.703 (0.263 to 1.143)	0.002
55–59	0.415 (−0.039 to 0.868)	0.073
60–64	0.127 (−0.349 to 0.602)	0.602
65–69	−0.008 (−0.49 to 0.474)	0.974
70–74	0.065 (−0.391 to 0.522)	0.779
75–79	0.257 (−0.17–0.683)	0.238
80–84	0.336 (−0.094 to 0.767)	0.126
85-89	0.307 (−0.17 to 0.784)	0.207
**Period**
1994	−0.113 (−0.404 to 0.177)	0.444
1999	−0.06 (−0.328 to 0.208)	0.662
2004	−0.086 (−0.352 to 0.18)	0.526
2009	−0.022 (−0.287 to 0.244)	0.872
2014	0.088 (0.024 to 0.148)	0.518
2019	0.193 (0.082 to 0.304)	0.158
**Cohort**
1909	0.267 (−0.745 to 1.279)	0.605
1914	0.304 (−0.419 to 1.027)	0.41
1919	0.373 (0.289 to 0.461)	0.022
1924	0.369 (−0.164 to 0.903)	0.175
1929	0.327 (−0.169 to 0.822)	0.196
1934	0.267 (−0.197 to 0.731)	0.259
1939	0.176 (0.048 to 0.313)	0.044
1944	0.118 (0.035 to 0.202)	0.037
1949	0.078 (−0.451 to 0.608)	0.772
1954	0.052 (−0.492 to 0.596)	0.851
1959	0.036 (−0.524 to 0.596)	0.9
1964	0.001 (−0.582 to 0.585)	0.997
1969	−0.067 (−0.686 to 0.551)	0.831
1974	−0.151 (−0.819 to 0.518)	0.659
1979	−0.221 (−0.958 to 0.517)	0.558
1984	−0.277 (−1.136 to 0.582)	0.528
1989	−0.328 (−1.387 to 0.731)	0.544
1994	−0.391 (−0.66 to −0.15)	0.005
1999	−0.445 (−0.53 to −0.37)	0.028
2004	−0.489 (−4.861 to 3.883)	0.826

**Figure 7:**
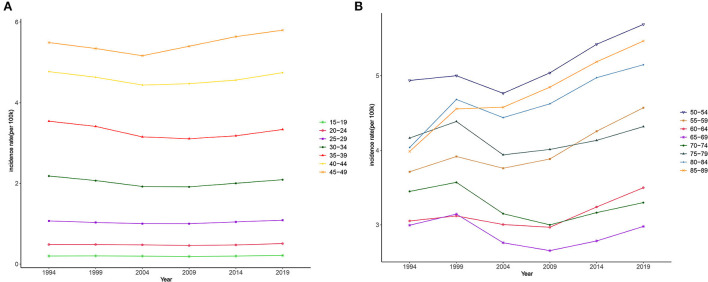
Period trends in the incidence of NAFLD. **(A)** Period trends of population aged 15–49. **(B)** Period trends of population aged 50–89.

Figure 7 shows how ASIR varies between periods. The changes in different age groups over different periods can be divided into two types. The first type was for those aged 15–49 years (Figure 7A). A typical characteristic of this type is that the ASIR of all groups changed little during the observation period; that is, the period effect is small. The other type was for those aged 50–89 years, which had overall trends that first decreased and then increased, especially after 2004; except for in those aged 65–69 and 70–74 years, the groups had significantly increasing trends (Figure 7B). Quantitative analysis using the APC model indicated that the period effect had an increasing trend over time. The effect values of the observation points in 2014 and 2019 were 0.088 and 0.193, respectively, and were statistically significant and gradually increasing (Table 2).

**Figure 8:**
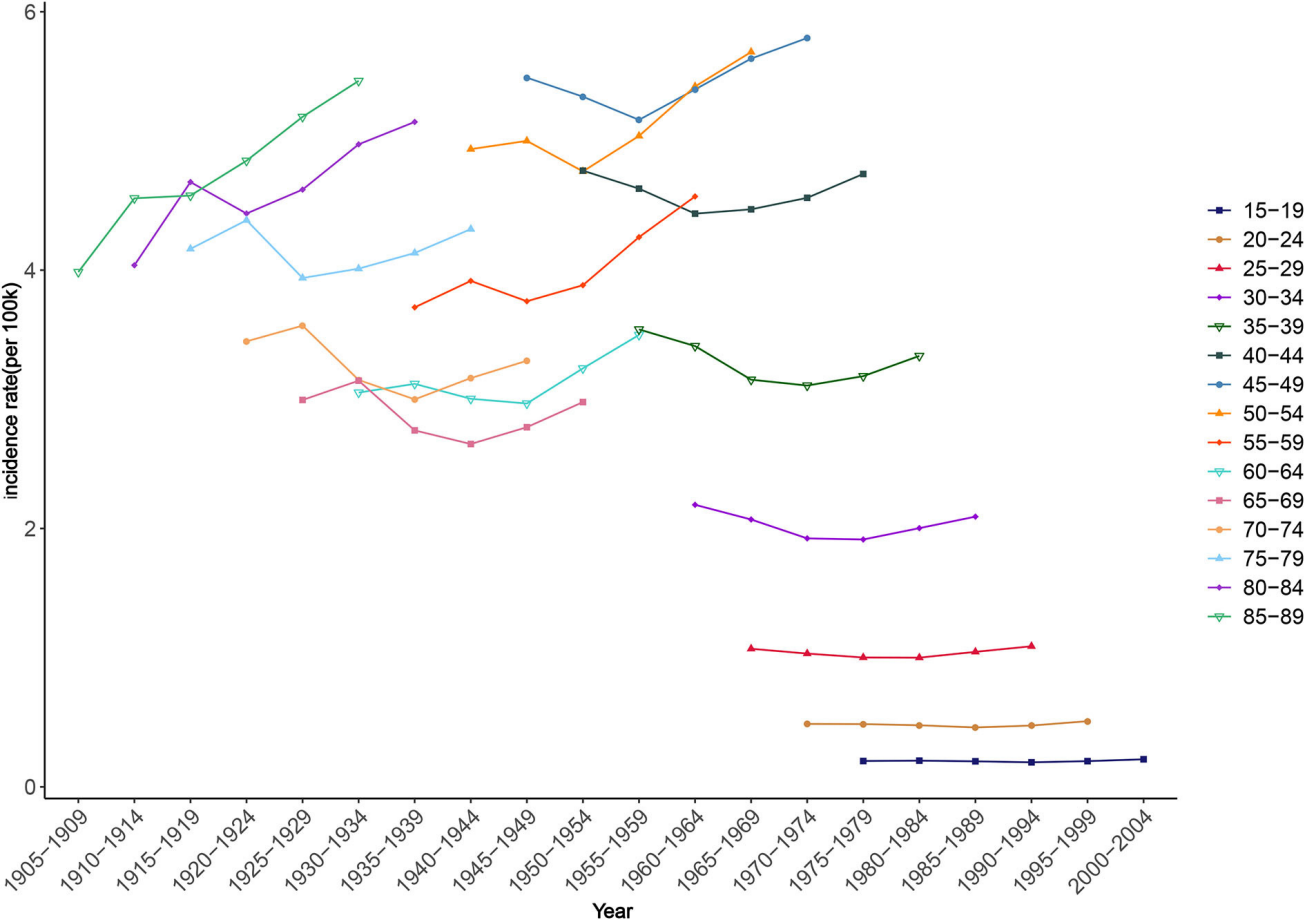
Cohort trends in NAFLD incidence.

The fluctuation amplitudes of the lines in Figure 8 provide a preliminary estimate of the cohort effect. Smaller fluctuations indicate smaller cohort effects. Younger groups had smaller influences from birth cohort factors on NAFLD risk. For example, in those aged 15–19 and 20–24 years, the incidence rates changed little during the observation period, suggesting that there were only small differences in ASIR among these age groups between birth cohorts. Among all the statistically significant cohort effects in the APC model, that of NAFLD risk for those born during 1915–1919 was 0.37, the highest among all birth cohorts, while the cohort effect had an overall decreasing trend during 1990–2019 (Table 2).

## Discussion

The acceleration of economic globalization has brought about significant changes in the methods of production and lifestyles. The area of public health and medical care is mostly reflected in changes in the spectrum of human diseases (26). Chronic diseases have become the main cause of death, disability, and quality-of-life deterioration worldwide. NAFLD is an emerging pandemic due to its huge burden on patients and impact on health systems (27–29). A previous study based on GBD2017 found that non-alcoholic fatty liver disease will gradually replace hepatitis B as the leading cause of cirrhosis, with the prevalence of compensatory cirrhosis and decompensated cirrhosis caused by NAFLD doubling and tripling, respectively, between 1990 and 2017 (30). According to GBD2019, the ASIR of cirrhosis caused by non-alcoholic fatty liver disease was 1.63 per 100,000 in 2019, compared with 1.49 per 100,000 in 1990. Cardiovascular-related death has also became the leading cause of death in NAFLD (31). Although NAFLD is a major cause of increasingly serious liver diseases and cardiovascular problems worldwide, many clinicians still have insufficient understanding of this condition, and even underestimate its incidence and associated risks (32, 33). In many countries there is also little awareness among policymakers of the trends of the NAFLD pandemic, resulting in a lack of detailed long-term plans (34). The present study therefore used representative and comprehensive data from the GBD study over a 29-year period to analyze the long-term trends of worldwide NAFLD incidence, in order to provide assistance for the challenging task of preventing and controlling it.

The ASIR of NAFLD remained stable over the 29-year study period, but the number of new cases in 2019 was almost double that in 1990, and NAFLD remains a public health problem that cannot be ignored in every area of the world. Previous studies have paid little attention to sex differences in NAFLD. It was particularly interesting that there seems to be a cutoff between the ages of 45 and 49 years, with males younger than these ages and females older than these ages having higher ASIR. We speculate that sex differences might be attributable to lifestyle and biological differences. Males consume more tobacco, alcohol, and processed meat, and they are more commonly overweight and obese, which are both factors closely related to NAFLD occurrence (35–37). Poor lifestyle may therefore be a reason for the higher ASIR in young males. On the other hand, estrogen, as an antioxidant, is considered to be a protective factor for NAFLD by inhibiting hepatic stellate cell proliferation, thus reducing the risk of intrahepatic fat accumulation and fibrosis (38, 39). Decreased estrogen levels leading to subcutaneous fat redistribution and visceral fat accumulation may therefore increase the NAFLD risk in postmenopausal females, which might also explain the increase in ASIR among females older than 45 years (40). Another interesting finding is that ASIR showed a unimodal distribution in 1990 and a bimodal distribution in 2019, with males and females consistent. This new growth trend may be linked to a sharp increase in the median age of the population as a result of increased life expectancy around the world (41, 42). Because NAFLD is a hepatic manifestation of metabolic syndrome, its risk factors tend to increase with age, and elderly patients tend to show more serious biochemical and histological changes (decline in hepatic blood flow and liver volume, accumulation of visceral fat) and have more metabolic risk factors (hypertension, diabetes, hyperlipidemia and obesity) (39, 43). The new prevalence of NAFLD in elder people means that clinicians in the future may need to consider early intervention for the disease earlier and optimize management of modifiable risk factors.

Latin America, which had the highest NAFLD incidence (and far higher than in other regions), faces a serious challenge. Previous studies suggest that Latin America is the region with the fastest growing population with type 2 diabetes worldwide, as well as with the highest obesity rates, which are both major risk factors for NAFLD (44, 45). Countries in Latin America also suffer from inadequate disease awareness, uneven health resource distribution, and a lack of long-term preventive action plans to address the rapid growth of NAFLD (46). Strengthening education and communication, raising public awareness, and developing sound disease classification criteria and long-term prevention plans (including for obesity and type 2 diabetes) are urgent priorities for Latin America to address their challenges with NAFLD (47, 48). NAFLD is a multifactorial disorder influenced by individual behaviors and genetics, as well as cultural, demographic, economic, medical, and environmental influences (49). The recent diet trends of excessive energy intake and sedentary lifestyles, and unbalanced medical resources can somewhat explain the differences among the five SDI subgroups (50, 51). However, it is worth noting that the GBD study has less data for the low- and low-middle-SDI groups, and so caution is required when considering the detected trends.

In the joinpoint model, the worldwide NAFLD incidence was analyzed according to time periods. There were five statistically significant turning points in the six time periods identified by the joinpoint model. ASIR showed an increasing trend overall, with an especially rapid increase after 2005. This increasing trend may be related to socioeconomic development, lifestyle changes, advances in medical technology, and increased awareness of NAFLD. Overall, this increasing trend was related to both the lifestyle changes brought about by the socioeconomic development described previously and the increased public awareness of NAFLD. In the early stages of NAFLD, patients often have no obvious clinical symptoms, and so a reliable diagnosis can only be obtained at a later stage (52). With the development of society and the improvement of the understanding of NAFLD among the general public, the gradual popularization of physical examinations, new biochemical indicators, scoring systems, and imaging results can be used to diagnose NAFLD and its stage, which helps to promote the detection of new cases (53, 54). It is worth noting that ASIR showed a downward trend from 1996 to 2005, and similar trends have been reported in previous studies (55). We speculate that this may be related to the insufficient understanding of NAFLD in the early stage, the definition and confirmation of cases of NAFLD hindered the epidemiological study of NAFLD to some extent in early years (56). Cryptogenic cirrhosis is the main alternative diagnosis of early NAFLD, and the early definition of NAFLD often included the ICD code of cryptogenic cirrhosis, thus overestimating the true ASIR of NAFLD (57–59). There was an initial improvement between 2000 and 2005, when NAFLD began to be recorded as an independent diagnosis, and between 2001 and 2005, the frequency of NAFLD as the primary diagnosis increased 35-fold (59). This more accurate recording may have led to a temporary decline in ASIR in NAFLD, which has since shown a new upward trend due to the prevalence of obesity, type 2 diabetes and poor lifestyles, as well as advances in screening.

Trend and quantitative analyses of the age effect found that the effect coefficient was negative for those younger than 30 years. Being young might be a protective factor for NAFLD, as the age effect increased with age, which was consistent with the conclusion of a previous large-scale population survey in some different regions (60, 61). Unsurprisingly, the age group with the greatest age effect was 40–49 years, as this group tends to be the main source of family income, and heavy work schedules leave little time for physical activity and other healthy lifestyle practices (62). The increase in the period effect revealed the increasing number of NAFLD patients. It is undeniable that the prevalence of obesity and type 2 diabetes caused by social environment changes and increasing social stress are likely to be causes of NAFLD. On the other hand, public awareness improvements and medical technology advancements, which enable more potential cases to be detected, are also potential factors for period effect increases (52). This suggests that reducing period effects, improving disease awareness, developing new cost-effective screening methods, and establishing reasonable risk stratification and medical approaches for NAFLD patients are not the only effective means to actively respond to the NAFLD pandemic (63).

Encouragingly, the cohort effect had a downward trend after excluding age and period effects; that is, the cohort effect can reduce NAFLD risk. This reflects the progress that has been made in the prevention of NAFLD in recent decades. For example, NAFLD-related risk factors such as food insecurity and mental stress have been discovered recently, and the diagnostic guidelines for NAFLD have been updated constantly, laying a foundation for its prevention (64). Meanwhile, many consensus opinions and survey reports on this issue have been formed in various regions. For example, experts from 29 European countries completed surveys on national NAFLD policies, guidelines, awareness, surveillance, diagnoses, and clinical assessments, and Latin America published a consensus on current challenges and opportunities, which undoubtedly contributed to the adoption of adequate public health policies (34, 46). The global establishment of primary preventive care for chronic metabolic diseases is also reducing the negative impact of NAFLD (65).

In face of the increasing burden of NAFLD, some reasonable prevention strategies will help reduce the pressure on the medical system and promote the rational allocation of resources. First, here is no doubt that the introduction and investment of a long-term plan for NAFLD will help curb the spread of NAFLD worldwide, but strategies and plans at the national and global levels are still lacking (66). Second, it is important to raise awareness of NAFLD among medical staff and the general public. Improved public awareness will facilitate the implementation of more comprehensive early screening and thus early intervention and management, which is very important for NAFLD, a reversible disease. In view of the low operability of liver biopsy and the low sensitivity of ultrasound diagnosis for patients with liver fat content <20%, the proposed new and more accurate diagnosis method will also be beneficial to the diagnosis of the disease (67). Specifically, a simple, effective information and de-stigmatizing terminology to describe disease risk factors and potential consequences is necessary. In the absence of a broader consensus, NAFLD is currently the name commonly used. A point of contention for the past two years has been the attention given to the term “metabolic dysfunction-associated fatty liver disease” (MAFLD) as a possible surrogate (68). An international panel of experts from 22 countries supported this initiative as MAFLD better reflects the etiology and metabolic basis of the disease (47). However, other experts have expressed concerns about prematurely changing the name without fully considering its broad implications, from diagnostic criteria to trial end points. This lack of consensus will undoubtedly reduce the level of attention for NAFLD, and terminology agreed upon by hepatologists, chronic metabolic disease specialists, community health care providers, policy makers and the general public needs to be proposed as soon as possible, because in addition to clinical and academic considerations, the more important question to address is how to raise awareness of the disease among the wider audience possible and avoid stigma, as NAFLD is often associated with stereotypes such as obesity and laziness (69, 70). Third, individualized early intervention is helpful. Active control of hypertension, hyperlipidemia and diabetes can help reduce the occurrence of NAFLD. Balanced diet and adequate physical activity are also low-cost and high-yield measures to prevent NAFLD. For men, reducing consumption of tobacco, red meat and a sedentary lifestyle may narrow the gender gap. Due to the changes of metabolic function and weak exercise ability, the elderly population has certain particularity. Personalized diet and exercise program may be a better strategy for early intervention.

While the data from the GBD database fill gaps in the study of the long-term trends of NAFLD incidence, there are some limitations in the interpretation of our results. Firstly, the accuracy of the results relied on estimates provided in the GBD study, which were calculated from many different data sources, and so the results need to be validated by large-scale epidemiological surveys. Secondly, endogenous factors cause unbiased estimations and a small variance, but its complex operation method leading to the parameter estimations was not intuitive and cannot explain the practical significance of the parameter estimations. Thirdly, the obtained data lacked NAFLD staging, screening, and other specific information, making further analysis difficult.

## Conclusion

This study has provided a comprehensive overview of the long-term trends in NAFLD incidence. NAFLD incidence varied greatly between ages, sexes, socioeconomic statuses, and geographical regions. Adult males, postmenopausal females, Latin American populations, and people in developing countries are at a high risk of developing NAFLD. In order to reduce its huge disease burden, screening of these populations should be increased in the future. Although prevention measures have recently achieved initial results, top priorities still include raising its public awareness, developing diagnostic criteria, identifying cost-effective screening methods, and seeking policy support to deal with NAFLD as a major public health problem in the future.

## Data availability statement

Publicly available datasets were analyzed in this study. This data can be found here: http://ghdx.healthdata.org/gbd-results-tool.

## Ethics statement

Ethical review and approval was not required for this study in accordance with the local legislation and institutional requirements.

## Author contributions

WW and AF analyzed the data and wrote the manuscript. DL, WM, SZ, FX, and DH acquired and analyzed the data. JL designed the study and participated in data analysis and interpretation. WW, WM, and AF are equal contributions to this work. All authors approved the paper.

## Conflict of interest

The authors declare that the research was conducted in the absence of any commercial or financial relationships that could be construed as a potential conflict of interest.

## Publisher's note

All claims expressed in this article are solely those of the authors and do not necessarily represent those of their affiliated organizations, or those of the publisher, the editors and the reviewers. Any product that may be evaluated in this article, or claim that may be made by its manufacturer, is not guaranteed or endorsed by the publisher.
